# Maximizing Athletic Performance Through Innovation, Education, and Practical Applications

**DOI:** 10.1007/s40279-017-0700-8

**Published:** 2017-03-22

**Authors:** Lawrence L. Spriet

**Affiliations:** Human Health and Nutritional Sciences, University of Guelph, Guelph, ON N1G 2W1 Canada

Maximizing athletic performance is a passion that athletes, coaches, athlete support professionals, and sports scientists share. A thorough understanding of the basics of all aspects of human physiology and the ability of the body to adapt to the environmental stress of exercise training is the foundation we use to explain the incredible athletic and sport performances that are commonplace in today’s world. This knowledge base is forever expanding through innovation and the gathering of information in laboratory testing and real-world field settings. Of equal importance is the fact that this information must be passed on to the athletic consumers through education and practical applications. The papers in this supplement examine some of the latest innovations, educational information, and practical applications in sports science and sports nutrition that aim to improve athletic performance. The Gatorade Sports Science Institute (GSSI) brought together researchers for a meeting in November 2015 to discuss a wide variety of relevant topics of interest in this area. Following the meeting, authors were asked to summarize the recent work in their topic area, resulting in the manuscripts in this supplement, which is the fourth supplement in Sports Medicine in this series.

Innovation and out-of-the-box thinking is critical for pushing back the boundaries of optimal human performance. One example is the work being done on engineering ligaments. A reality of competing in sports is that ligament injuries occur and understanding more about how we can maximize repair and decrease the time to return to play will have widespread usefulness. This information may also help in the normal recovery from a hard workout or game, as the recovery period is also the start of preparation for the next practice or competition, and often the time is short. This supplement also examines two areas of innovation in the processing or metabolism of fuels during exercise. It is becoming clear that our understanding of the energy-producing events in the mitochondria is far more complex than originally believed. Evidence is emerging that there are many ways to regulate energy production in the mitochondria independent of changes in mitochondrial content. It makes sense that adenosine diphosphate (ADP), a major breakdown product of energy use (adenosine triphosphate, ATP) in the cell, would play a large role in stimulating further production of ATP and regulating the movement of ATP and ADP across the mitochondrial membranes. Fructose is an example of a fuel we consume that has not been thoroughly studied or appreciated. The energy-producing potential of fructose is similar to glucose but there are many unique aspects of its metabolism we need to understand to realize the full potential of fructose as an exercise fuel.

The section on education in this supplement examines attempts to pull together and update the field in three distinct areas: exertional rhabdomyolysis, periodized nutrition, and the ‘critical power’ concept. All three papers aim to provide athletes, coaches, and support personnel with information to tailor their approach to training and competitions. In sports where the exercise effort is intense and/or long in duration, there are limits to what the contracting muscles can handle before damage can become concerning. Preventing these situations with the available knowledge will help keep athletes healthy and safe. Proper nutrition for athletic performance is now routinely seen as a part of every athlete’s preparation, but we are still experimenting with the timing of fuel ingestion to maximize the adaptation to and recovery from exercise and to ultimately maximize performance. The periodized nutrition concept explores what is known and not known in this area. The paper on ‘critical power’ explores how this concept may be applied to sports that employ repeated bouts of high-intensity intermittent efforts. The critical power output is that point between power outputs that can be sustained for some time and those where exercise intolerance quickly occurs. The authors extend the application of this concept from endurance-type sports to the study of the physiological responses, fatigue mechanisms, and performance capacities during team sports (e.g., basketball, football, hockey, rugby).

The final papers in this supplement aim to provide the reader with practical information regarding the application of what is known in the areas of performance supplements, the gut as an athletic organ, and the importance of hydration during athletic endeavors. Each of these papers emphasize logical approaches to using the existing information to decide when and how to use performance supplements, train the gut, and control hydration to maximize training and competition performance. Athletes, coaches, and practitioners can use this information directly in their day-to-day decisions.

A critical aspect of this supplement is the attention to evidence-based information as it relates to maximizing athletic performance. It is important to push the limits of what we know, at first conceptually, and then with well designed approaches to determine if there is merit in the application of the concept to the real athletic/sports world.

Lawrence L. Spriet, PhD

Guest Editor

